# *Euscorpiops lii* sp. nov. and a key of the genus *Euscorpiops* Vachon, 1980 (Scorpiones, Scorpiopidae) from China

**DOI:** 10.3897/zookeys.968.48723

**Published:** 2020-09-16

**Authors:** Zhiyong Di, Sha Qiao

**Affiliations:** 1 College of Life Science, Institute of Life Science and Green Development, Hebei University, Baoding 071002, Hebei, China Hebei University Baoding China

**Keywords:** new species, scorpion, Tibet, Xizang

## Abstract

A new species, *Euscorpiops lii***sp. nov.**, from Xizang (Tibet) in southwest China is described herein. Adult scorpions in this species are principally characterized by yellow-brown colour, a length of less than 40 mm, 17 trichobothria on the external surface of the pedipalp patella and usually six trichobothria on the ventral surface of the pedipalp patella in both sexes. With the description of this new species, the number of known species of the genus *Euscorpiops* from China is raised to 13 (five species found in Xizang, including the new species, and eight other species in Yunnan). A key to the species of the genus *Euscorpiops* from China is presented.

## Introduction

The genus *Euscorpiops* has medium size scorpions in the family Scorpiopidae. Scorpions of this genus are distributed in South and Southeast Asia, including 27 species inhabiting the Oriental region from Pakistan to Vietnam (Kovařík et al. 2015; Ythier 2019). In China, the species of genus *Euscorpiops* were found in Xizang and Yunnan. Di et al. (2010) recorded ten species of *Euscorpiops* from China: *E. asthenurus* (Xizang), *E. kamengensis* (Xizang), *E. karschi* (Xizang), *E. novaki* (Xizang), *E. puerensis* (Yunnan), *E. shidian* (Yunnan), *E. vachoni* (Yunnan), *E. validus* (Yunnan), *E. yangi* (Yunnan), and *E. xui* (Yunnan). Di et al. (2011) added *E. kubani* to the fauna of Yunnan. Di et al. (2013–2015) revised the fauna of the genus *Euscorpiops* in Xizang and provided the history of study of this genus, an updated checklist and the distribution and key of the order Scorpiones in China. Kovařík et al. (2015) revised the checklist and key of the genus *Euscorpiops* including eleven species distributing in China. Ythier (2019) described a new species *E. zhangshuyuani* found in Yunnan. Until now, 13 species of this genus are currently recognized as present in China (including the new species).

## Materials and methods

Illustrations and measurements were produced using a Leica M205 stereomicroscope. Measurements followed Sissom (1990) and are given in mm. Trichobothrial notations followed Vachon (1974), and the morphological terminology mostly followed Hjelle (1990). The terminology of metasomal carination followed Vachon (1952), and the terminology of pedipalp chelal carinae followed Soleglad and Sissom (2001). Type series of the new species are deposited in the Museum of Hebei University, Baoding, China (MHBU).

## Taxonomic treatment

### Family Scorpiopidae Kraepelin, 1905

**Genus *Euscorpiops* Vachon, 1980**

#### Euscorpiops lii sp. nov.

Taxon classificationAnimaliaScorpionesEuscorpiidae

C50DAF52-F2BD-5AE4-905A-27E1C4C6801E

http://zoobank.org/7C8D77C3-DD77-4E6F-ACAE-887BB8E2ABD4

Figures 1–4 , 5–16 , 17–24 , 25–30 , Table 1

##### Type material.

Male holotype, China: Xizang, Longzi County (Lhünzê County), Zhiyong Di, Kai Wang, Jia Xiang & Dezheng Meng leg, (Ar.-MHBU-XZLZ1901); 6 male and 13 female paratypes (including 1 male and 2 female immatures) (Ar.- MHBU-XZLZ1903–08, Ar.- MHBU-XZLZ1902, 09–20), same location data as holotype.

##### Diagnosis.

*Euscorpiops lii* sp. nov. differs from all other species in the genus on the basis of the following combination of characters: yellow-brown colour, small size (length of adults less than 40.0 mm), 17 trichobothria on the external surface of pedipalp patella (5 *eb*, 2 *esb*, 2 *em*, 4 *est*, 4 *et*) and six or seven (usually six) trichobothria on the ventral surface of pedipalp patella, chela with an average length/width ratio of 3.7 in males (3.6–3.8, six adults) and 3.3 in females (3.2–3.4, six adults), pedipalp chela fingers on adult males and females scalloped, and pectinal teeth count 4–6 with five or six in males (usually six) and 4–6 (usually five) in females, pectinal fulcra present.

##### Etymology.

Patronym in honour of Prof. Wenxin Li (Wuhan University), who greatly contributed to the research on scorpion toxins and genome.

##### Description of the holotype.

**Figures 1–4.**
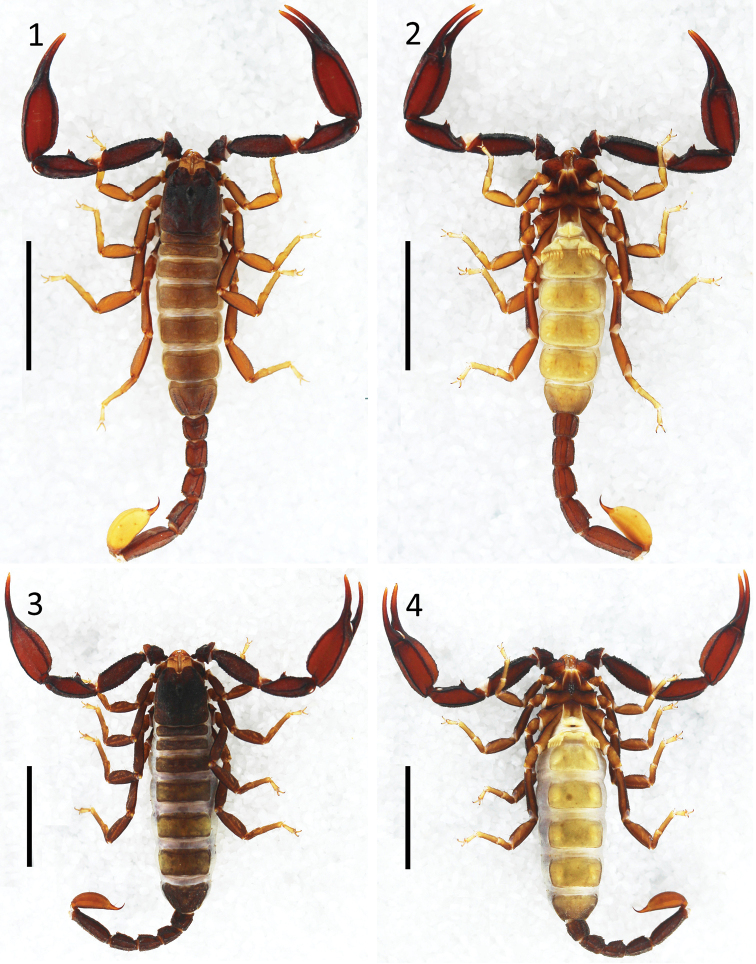
Habitus of *E. lii* sp. nov. **1, 2** male holotype (Ar.-MHBU-XZLZ1901), dorsal and ventral views **3, 4** female paratype (Ar.-MHBU-XZLZ1902), dorsal and ventral views. Scale bars: 10.0 mm.

***Coloration*** (Figs 1, 2): Carapace, yellow-brown. Median and lateral ocular tubercles black-brown. Tergites and metasomal segments yellow-brown, while as metasoma with black-brown carinae; vesicle yellow, with a brown aculeus. Chelicerae yellow, with darker, yellow-brown fingers. Pedipalp yellow-brown, with black-brown carinae. Legs yellow. Sternum, yellow-brown. Genital operculum pectines, and sternites pale yellow.

***Morphology.*** The integument is coarse for the carapace, tergites, metasomal segments, legs and pedipalps while the integument smooth for the coxapophysis, coxae, sternum, genital operculum, pectines, and sternites.

**Figures 5–16.**
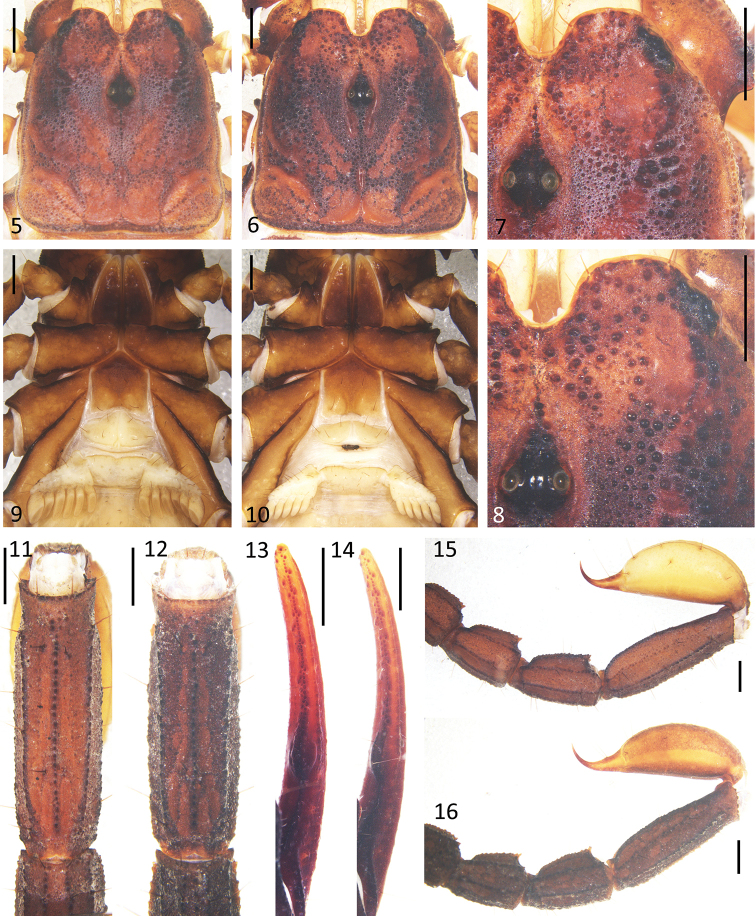
*E. lii* sp. nov. **5, 7, 9, 11, 13, 15** male holotype (Ar.-MHBU-XZLZ1901) **6, 8, 10, 12, 14, 16** female paratype (Ar.-MHBU-XZLZ1902) **5, 6** carapace **7, 8** eyes and nearby area **9, 10** genital operculum and pectines **11, 12** metasomal segment V, ventral surface **13, 14** dentate margin of movable finger, showing rows of granules **15, 16** metasomal segments III–V and telson, ventral surface. Scale bars: 1.0 mm.

***Prosoma*** (Figs 5, 7, 8): Carapace with sparse, fine granules; lateral furrow, broad; anterior median furrow, broad and deep; posterior median furrow, deep; margin behind lateral eyes with granules, other margins smooth. Median eyes situated anteriorly compared to centre of carapace; three pairs of lateral ocelli with posterior-most one the smallest. Median ocular tubercle, coarse with granules and a median furrow. Lateral ocular tubercle with some granules around lateral eyes.

***Mesosoma***: Tergites densely covered with fine granules; tergite II to tergite VI with a median carina; tergite VII with two pairs of lateral carinae with big granules. Pectinal teeth count 6/6, fulcra small (Fig. 9). Genital operculum subtriangular with genital papillae protruding (Fig. 9). Sternites smooth and shiny; segment VII with some big granules and four weak ventral carinae.

***Metasoma***: Segments II to V longer than wide; segments I to V with respectively 10-8-8-8-7 granular carinae; segment V with a pair of vestigial lateral carinae; all dorsal carinae crenulated, slightly stronger distally (Figs 11, 15). Vesicle with sparse weak granules and few setae (Fig. 15).

**Figures 17–24.**
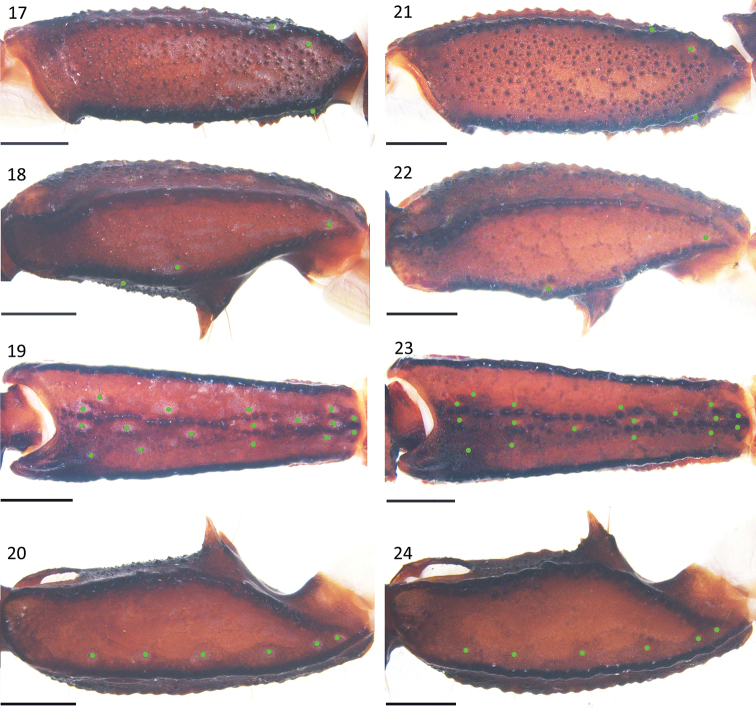
*E. lii* sp. nov. **17–20** male holotype (Ar.-MHBU-XZLZ1901) **21–24** female paratype (Ar.-MHBU-XZLZ1902) **17, 21** femur dorsal surface **18–20, 22–24** patella dorsal, external and ventral surfaces. Scale bars: 1.0 mm.

**Figures 25–30.**
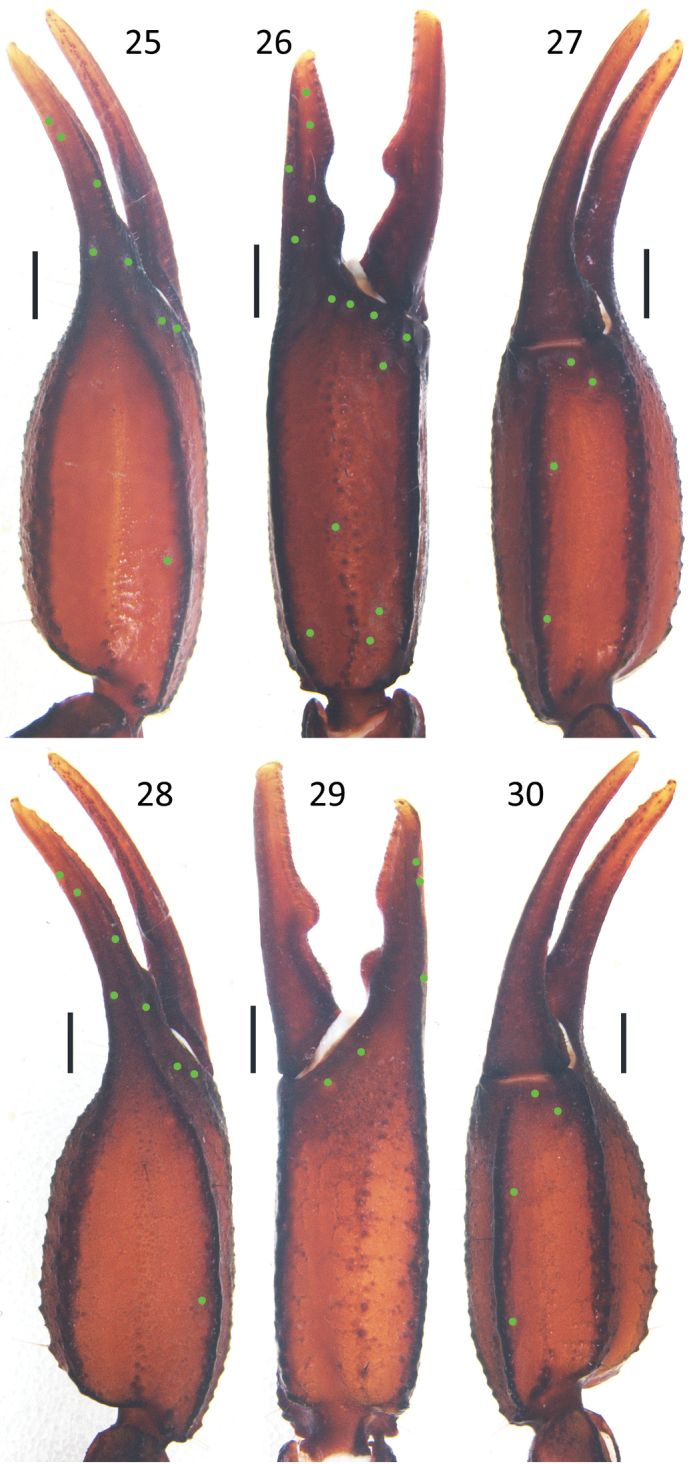
*E. lii* sp. nov. **25–27** male holotype (Ar.-MHBU-XZLZ1901), chela dorsal, external, and ventral surfaces **28–30** female paratype (Ar.-MHBU-XZLZ1902), chela dorsal, internal and ventral surfaces. Scale bars: 1.0 mm.

***Pedipalps***: Femur with external, dorsointernal, dorsoexternal, ventrointernal, ventroexternal, and internal carinae with big granules; the integument with scattered granules dorsally and smooth ventrally (Fig. 17). Patella with big granules on the dorsointernal, dorsoexternal, ventrointernal, ventroexternal, and external carinae; two large spinoid granules present on the internal surface; the integument with scattered granules dorsally and ventrally (Figs 18–20). Trichobothrial pattern C, neobothriotaxic (Vachon, 1974); patella with 17 external trichobothria (5*eb*, 2 *esb*, 2 *em*, 4 *est*, 4 *et*), 6 (right) and 7 (left) ventral trichobothria (Figs 19, 20). Chela with granules forming indistinct reticulated pattern, all carinae granular and coalesced except the dorsal secondary, dorsointernal, and ventromedian carinae vestigial; dorsointernal carina just with some sparse big granules, movable fingers and fixed fingers with scalloped margins, a pronounced lobe in the movable finger and a corresponding notch in the fixed fingers (Figs 13, 25–27).

***Legs***: Trochanters with few setae. Femora dorsal surfaces with some small granules, externally with one granular carina, internally with two granular carinae. Patellae externally with one granular carina and internally with one dentate carina. Tibiae with few setae, without spurs. Basitarsi with few setae and some short spinules terminally and two lateral pedal spurs. Tarsi ventrally with a row of short and strong spinules. Tarsal ungues curved and hook-like.

##### Variation.

Morphology of both adult sexes are similar to holotype (Figs 3, 4). Colouration of adult female is darker than males (Figs 1–4): Carapace, tergites, and metasoma dark yellow-brown; vesicle and legs yellow-brown, legs with yellow tarsi. Feature figures of adult females are provided (Figs 6, 10, 12, 14, 16, 21–24, 28–30). Chela with an average length/width ratio of 3.7 in males (3.6–3.8, six adults) and 3.3 in females (3.2–3.4, six adults). Pedipalp patella with six or seven (usually six) trichobothria on its ventral surface. Pectinal teeth count 4–6 with five or six in males (usually six) and 4–6 (usually five) in females. The adults have more pronounced lobes on the movable fingers of the chela and a more pronounced notch in the fixed finger compared with immature and juvenile individuals of both sexes. Measurements in Table 1. Feature datasets in Table 2.

**Table 1.** Measurements (mm) of *Euscorpiops lii* sp. nov. holotype (male, Ar.-MHBU-XZLZ1901) and paratype (female, Ar.- MHBU-XZLZ1902).

	Holotype ♂	Paratype ♀
Total length:	37.4	38.8
Carapace:		
-Length	5.1	5.7
-Anterior width	2.9	3.4
-Posterior width	4.7	5.3
Mesosomal segments:		
-Length	11.8	13.7
Metasomal segment I:		
-Length	2.0	2.1
-Width	2.0	2.1
-Depth	1.8	1.7
Metasomal segment II:		
-Length	2.3	2.3
-Width	1.9	1.9
-Depth	1.7	1.6
Metasomal segment III:		
-Length	2.5	2.3
-Width	1.8	1.7
-Depth	1.7	1.7
Metasomal segment IV:		
-Length	3.0	2.9
-Width	1.7	1.5
-Depth	1.7	1.7
Metasomal segment V:		
-Length	5.0	4.6
-Width	1.6	1.6
-Depth	1.6	1.4
Telson:		
-Length	5.7	5.2
-Width	1.9	1.7
-Depth	2.0	1.5
Pedipalp femur:		
-Length	5.3	6.0
-Width	1.8	2.1
-Depth	1.4	1.6
Pedipalp patella:		
-Length	4.7	5.1
-Width	2.1	2.6
-Depth	1.8	2.0
Chela:		
-Length	10.1	10.8
-Width (manus)	2.8	3.2
-Depth (manus)	2.2	2.5
Movable finger:		
-Length	5.2	6.0
Pectinal teeth (left/right)	6/6	5/5

##### Habitat.

Found under stones in mountain boscage in Longzi County [28°25'N, 92°57'E], 3104 m elevation.

##### Known distribution.

Known only from Longzi County, Xizang Autonomous Region (Tibet), southwest China.

##### Relationships.

The new species appears to be related to the other species of the genus *Euscorpiops* from Xizang: *E. asthenurus* and *E. kamengensis*. These are also the geographically closest species, but these species can be readily distinguished on the basis of the following combination of characters: (i) 17 trichobothria on the external surface of pedipalp patella (5 *eb*, 2 *esb*, 2 *em*, 4 *est*, 4 *et*) in *E. lii* sp. nov. (seven males and 13 females), 18 (5/2/2/4/5) in *E. asthenurus* (two males and one female) and 19 (5/2/2/5/5) in *E. kamengensis* (two females); (ii) six or seven (usually six) trichobothria on the ventral surface of the pedipalp patella in *E. lii* sp. nov., while there are eight or nine in *E. asthenurus*, and seven in *E. kamengensis*.

The following main features can be used to distinguish *E. lii* sp. nov. from the other more geographically distant species of the genus *Euscorpiops* occurring in Xizang, *E. karschi* and *E. novaki*: (i) length of adults less than 40.0 mm in *E. lii* sp. nov. (seven males and 13 females) while the length of adults more than 45.0 mm in *E. karschi* (one male and one female) and *E. novaki* (one male); (ii) the pedipalp patella with 18 (5/2/2/4/5) external trichobothria in *E. karschi* (one male and one female) and 19 (5/2/2/5/5) in *E. novaki* (one male); (iii) the pedipalp patella with eight or nine ventral trichobothria in *E. karschi* (one male and one female) and nine in *E. novaki* (one male); (iv) the pectinal teeth count 4–6 with five, six (usually six) in males and 4–6 (usually five) in females in *E. lii* sp. nov., 7–9 in *E. karschi* (7–8 in three females, 8–9 in two males) and eight in *E. novaki* (one male).

**Table 2.** Feature datasets of species of *Euscorpiops* from China. BC, basic colour; BL, body length (mm); CL, chela length (mm, in one specimen); CW, chela width (mm, in the same specimen with CL); CS, chela shape; ETPP, external trichobothria of pedipalp patella (*eb*/*esb*/*em*/*est*/*et*); FS, fingers scalloped or non-scalloped (nearly straight); H, holotype; LW, length/width ratio of chela; PT, pectinal teeth count; VTPP, ventral trichobothria of pedipalp patella. L, locality; R, rarely; RE, references; S, Sex; U, usually; X, Xizang; Y, Yunnan.

Species	S	BL	BC	CL	CW	LW	CS	FS	VTPP	ETPP	PT	L	RE
*E. asthenurus*	♂	35.8–45	?	10.9	3.4	3.2	?	scalloped	8,9	18: 5/2/2/4/5	6,7	X	Kovařík 2000, 2015
	♀			15.4	3.9	3.9		slightly scalloped			5,6		
*E. kamengensis*	♀H	42.8	?	?	?	?	?	?	7	19: 5/2/2/5/5	4,5	X	Bastawade 2006
*E. karschi*	♂	45.1	yellow-brown	13.5	3.9	3.5	flattened dorsoventrally	scalloped	8	18$: 5/2/2/4/5	8,9	X	Di and Zhu 2009; Qi et al. 2005
	♀H	48.2		16.3	4.6	3.5			9		7,8		
*E. kubani*	♂	39–47	reddish-black	14.5	4.6	2.9–3.2	flattened dorsoventrally	scalloped	9,10	17–19(U18): 5,6/2/2/4/5	6–8(R6)	Y	Di et al. 2011; Kovařík 2004
	♀	43.6–48		13.5	4.4	2.7–3.2		slightly scalloped			6,7		
*E. lii*	♂H	37.4	yellow-brown	10.1	2.8	3.6–3.8	flattened dorsoventrally	scalloped	6,7(U6)	17: 5/2/2/4/4	5,6(U6)	X	this paper
	♀	38.8		10.8	3.2	3.2–3.4					4–6(U5)		
*E. novaki*	♂H	47	reddish-brown	16.2	4.5	3.6	flattened dorsoventrally	scalloped	9	19: 5/2/2/5/5	8	X	Kovařík 2005
*E. puerensis*	♂	57.1	dark red-brown to dark-brown	16.1	6.1	2.6–2.8	flattened dorsoventrally	scalloped	10,11	18: 5/2/2/4/5	7,8(U8)	Y	Di et al. 2010b
	♀ H	60		16.1	6.2						7,8(U7)		
*E. shidian*	♂H	47–60	dark red-brown	16.0	4.7	3.2–3.5	flattened dorsoventrally	non-scalloped	10–12(U11)	18$: 5/2/2/4/5	6–8(R6)	Y	Di et al. 2011; Qi et al. 2005
	♀	45–59.8		16.5	4.7								
*E. vachoni*	♂H	52.9	yellow-brown	?	5.6	<3.0	stout and rounded ?	scalloped	10	18$: 5/2/2/4/5	7,8	Y	Di et al. 2011; Qi et al. 2005
	♀	42.3#		?	3.3			slightly scalloped	?		7		
*E. validus*	♂H	50.0–59.8	dark-brown	18.5	6.2	2.9–3.2	flattened dorsoventrally	scalloped	8–11 (U9&10)	17,U18: 5/2/2/4/5	6–8(U6)	Y	Di et al. 2010a, 2011
	♀			19.0	6.1								
*E. xui*	♂	54–56	dark-brown	18.9	4.6	4.0–4.1	flattened dorsoventrally	non-scalloped	10	18,19: 5,6/2/2/4/5	8	Y	Di et al. 2011; Sun and Zhu 2010
	♀H	58–66		18.2	5.1	3.4–3.6					7		
*E. yangi*	♂H	47.8	dark-brown	14.6	4.2	3.4	flattened dorsoventrally	non-scalloped	9,10	18: 5/2/2/4/5	6,7	Y	Zhu et al. 2007
	♀	51.3		14.5	4.5	3.3					5,6		
*E. zhangshuyuani*	♂	?	yellowish-brown to reddish-brown*	?	?	>4.3	flattened dorsoventrally	?	?	?	?	Y	Ythier 2019
	♀H	49.1		16.8	3.9	4.2–4.3		slightly scalloped	11	18: 5/2/2/4/5	7,8		

“*” The colouration description did not seem to reflect much that of the photos of the specimens. “#” It maybe an immature. “?” There was no information or dubious description provided in papers. “$” It was recorded as 17 external trichobothria of pedipalp patella in the original description by Qi et al., (2005).

### Key to species of the genus *Euscorpiops* from China

**Figure 31.**
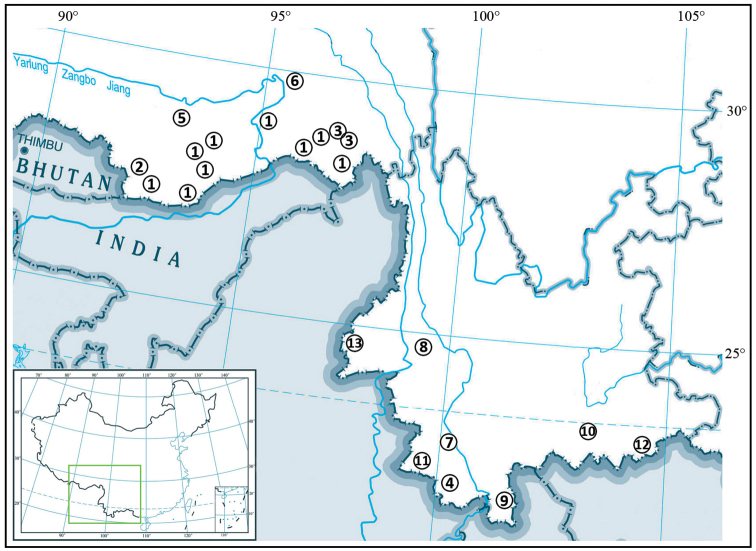
Map of the known distribution of *Euscorpiops* species from China (Xizang and Yunnan): **1***E. asthenurus***2***E. kamengensis***3***E. karschi***4***E. kubani***5***E. lii* sp. nov. **6***E. novaki***7***E. puerensis***8***E. shidian***9***E. vachoni***10***E. validus***11***E. xui***12***E. yangi***13***E. zhangshuyuani*.

(distribution map and feature datasets of species of *Euscorpiops* from China given in Fig. 31 and Table 2.)

**Table:** 

1	Length of adults less than 45.0 mm or more than 45.0 mm with light body colour (yellow-brown or reddish brown)	**2**
–	Length of adults more than 45.0 mm, with dark body colour (dark red-brown or dark brown, near black) or with stout and rounded chela	**6**
2	Pedipalp patella with 17 external trichobothria	***E. lii* sp. nov.**
–	Pedipalp patella with 18 or 19 external trichobothria	**3**
3	Pedipalp patella with 18 external trichobothria	**4**
–	Pedipalp patella with 19 external trichobothria	**5**
4	Dark blackish, length of adults less than 45.0 mm, pectinal teeth 5–7	***E. asthenurus***
–	Yellow-brown, length of adults more than 45.0 mm, pectinal teeth 7–9	***E. karschi***
5	Length of adults less than 45.0 mm, pectinal teeth 4 or 5	***E. kamengensis***
–	Length of adults more than 45.0 mm, pectinal teeth 8	***E. novaki***
6	Chela manus stout and rounded	***E. vachoni***
–	Chela manus flattened dorsoventrally	**7**
7	Chela length/width ratio less than 4 in male adults	**8**
–	Chela length/width ratio more than 4 in male adults	**12**
8	Chela length more than 17.0 mm	***E. validus***
–	Chela length less than 17.0 mm	**9**
9	Pedipalp chela fingers with non-scalloped (nearly straight) margins in male adults	**10**
–	Pedipalp chela fingers with scalloped margins in male adults	**11**
10	Dark red-brown, pedipalp patella with 10–12 (usually 11) ventral trichobothria	***E. shidian***
–	Dark brown, pedipalp patella with 9 or 10 ventral trichobothria	***E. yangi***
11	Length of adults less than 50.0 mm, chela length less than 15 mm	***E. kubani***
–	Length of adults more than 50.0 mm, chela length more than 15 mm	***E. puerensis***
12	Chela length/width ratio less than 4 in female adults	***E. xui***
–	Chela length/width ratio more than 4 in female adults	***E. zhangshuyuani***

## Supplementary Material

XML Treatment for Euscorpiops lii
